# Clinical analysis of 48 cases of malignant superior vena cava syndrome

**DOI:** 10.1186/s12957-021-02300-8

**Published:** 2021-06-23

**Authors:** Manzhen Sun, Xiaoli Chen, Hongfei Li, Xudong Zhang, Xiaofei Wang, Ruipan Zheng, Guowen Li, Lin Wang, Dianyuan Li

**Affiliations:** 1 School of Basic Medical Sciences, Zhengzhou University, No. 100 Science Avenue, Zhengzhou, 450000 Henan Province China; 2 Radiotherapy Inpatient Ward II, The First Affiliated Hospital of Zhengzhou University, No. 1 Eastern Jianshe Road, Zhengzhou, 450052 Henan Province China

**Keywords:** Superior vena cava syndrome, Malignant etiology, Radiotherapy, Chemotherapy, Comprehensive treatment

## Abstract

**Background:**

The aim of our study was to observe and compare the curative effect of radiotherapy, chemotherapy, and combined radiotherapy and chemotherapy, as well as comprehensive treatment on superior vena cava syndrome (SVCS) caused by malignant etiology.

**Methods:**

A total of 48 patients with malignant SVCS admitted to our hospital from 2015 to 2020 were selected in this study. According to the different treatment methods, they were divided into radiotherapy group (group 1, 10 cases), chemotherapy group (group 2, 8 cases), combined radiotherapy and chemotherapy group (group 3, 22 cases), and comprehensive treatment group (group 4, 8 cases).

**Results:**

There were no significant differences in efficacy and side effects among the four groups (all P > 0.05). Group 4 (median survival time of 36 months) could provide longer survival time than groups 1, 2, and 3 (median survival time of 10 months, 13.5 months, and 12 months, respectively).

**Conclusions:**

For patients with severe symptoms or good prognosis, comprehensive treatment could be selected to improve the quality of life and prolong the survival period; for patients with mild symptoms, radiotherapy, chemotherapy, or combined radiotherapy and chemotherapy could also reduce the symptoms of SVCS and treat tumor lesions.

## Background

Schechter found that about half of superior vena cava syndrome (SVCS) patients were caused by syphilis aneurysm and tuberculous mediastinitis [1]. With the development of medical technology, the main etiology of SVCS has changed from infectious factors to malignant tumor and some benign etiologies, including thrombosis and stenosis of central venous and pacemaker catheters [2]. At present, about 90% of SVCS is caused by malignant tumors (About 75% is lung cancer, and due to anatomy, right lung cancer is more likely to cause SVCS than left lung cancer. In addition, 80% of lung cancer patients are non-small cell lung cancer (NSCLC), but the incidence of small cell lung cancer (SCLC) is five times more likely to cause SVCS than that of NSCLC because of its location; about 15% is lymphoma, most of which are non-Hodgkin’s lymphoma (NHL)) [2–7]. Besides, about 5% of other causes include metastatic tumor, thymoma, thyroid cancer, esophageal cancer, germinoma, and breast cancer [3]. SVCS is caused by lumen stenosis or obstruction due to tumor compression and invasion of superior vena cava (SVC) or tumor thrombogenesis. In our study, only SVCS caused by malignant tumors was discussed. SVCS is a medical emergency, if not actively treated, the prognosis of SVCS caused by malignant tumors is often poor, and NHL patients tend to have a higher cure rate and a longer survival period [8, 9]. At present, glucocorticoids, diuretics, and endovascular treatment are often used to relieve symptoms, while radiotherapy, chemotherapy, combined radiotherapy and chemotherapy, and surgery are used for the final treatment [10, 11]. In this study, a retrospective analysis was performed to investigate the efficacy of radiotherapy, chemotherapy, and combined radiotherapy and chemotherapy, as well as comprehensive treatment on SVCS.

## Methods

### General data

Patients admitted to our hospital from 2015 to 2020 were selected to determine whether they had SVCS according to CT images and clinical symptoms. A total of 48 patients were enrolled according to the inclusion and exclusion criteria, and all of them had SVCS caused by malignant etiology. The ratio of male to female was 17:7, and the mean age was 56.68 years with extremes of 23 and 75 years. According to the results of anatomopathological examination, there were 20 cases of SCLC, 15 cases of NSCLC, 2 cases of NHL, and 9 cases of other tumors. According to the different treatment methods, the patients were divided into four groups, radiotherapy group (group 1, 10 cases), chemotherapy group (group 2, 8 cases), combined radiotherapy and chemotherapy group (group 3, 22 cases), and comprehensive treatment group (group 4, 8 cases) (see Table 1). Group 4 adopted radiotherapy, chemotherapy, SVC angiography, and stent implantation. This study was approved by the Ethics Committee of our hospital.

**Table 1:** General information (n)

	Group 1	Group 2	Group 3	Group 4
Gender				
Male/female	4/6	5/3	18/4	7/1
Histologic type				
SCLC	5	4	9	2
NSCLC	3	2	8	2
NHL	0	0	2	0
Others	2	2	3	4

Others include thymoma, mediastinal sarcoma, and mediastinal lung small cell carcinoma. *Group 1* radiotherapy group, *Group 2* chemotherapy group, *Group 3* combined radiotherapy and chemotherapy group, *Group 4* comprehensive treatment group, *SCLC* small cell lung cancer, *NSCLC* non-small cell lung cancer, *NHL* non-Hodgkin’s lymphoma

### Inclusion and exclusion criteria

Inclusion criteria are as follows: (1) Patients who were examined by CT, with lesions invading the SVC, and had SVCS symptoms; (2) patients with SVC compression syndrome for the first time; (3) patients without contraindications to radiotherapy; and (4) patients with malignant tumor confirmed by pathological examination.

Exclusion criteria are as follows: (1) Patients who also suffered from other tumors, (2) patients whose expected survival time was less than 3 months, and (3) patients who had SVCS caused by benign causes.

### Clinical manifestations

Among the 48 patients, there were 39 cases (81.25%) with facial and neck edema, 14 cases (29.17%) with upper limb edema, 4 cases (8.33%) with hoarseness, 7 cases (14.58%) with cervicothoracic varices, 13 cases (27.08%) with cough, 5 cases (10.42%) with expectoration, 14 cases (29.17%) with chest tightness, and 1 case (2.08%) with Horner syndrome (see Table 2).

**Table 2:** Clinical manifestations (n)

	Group 1	Group 2	Group 3	Group 4
Facial and neck edema	9	8	14	8
Upper limb edema	3	3	6	2
Hoarseness	1	0	3	0
Cervicothoracic varices	1	2	2	2
Cough	1	1	10	1
Expectoration	0	0	4	1
Chest tightness	3	2	7	2
Horner syndrome	1	0	0	0

Note: *Group 1* radiotherapy group, *Group 2* chemotherapy group, *Group 3* combined radiotherapy and chemotherapy group, *Group 4* comprehensive treatment group

### Evaluation criterion

According to the WHO criteria [12] for evaluating solid tumors, it is classified as complete remission (CR), the tumor completely disappears for more than 1 month; partial remission (PR), the product of the maximum diameter and the maximum vertical diameter of the tumor decreases by 50%, and other lesions do not increase, lasting for more than 1 month; stable degree (SD), the product of the maximum diameter and the maximum vertical diameter of the tumor decreases by no more than 50% and increases by no more than 25%, lasting for more than 1 month; progression degree (PD), the product of maximum diameter and maximum vertical diameter of tumor increases by more than 25%.

### Treatment methods

Patients in group 1 included lung cancer and mediastinal tumor, and the regimens included a total dose of more than 50 Gy (2 Gy/time) and 45 Gy (3 Gy/time). Platinums such as gemcitabine, etoposide, and docetaxel were used in group 2. In group 3, platinums such as etoposide, docetaxel, gemcitabine, and paclitaxel were used. The dose of radiotherapy in enrolled patients varied according to tissue types. NHL was treated with 38 Gy (1.8 Gy/time); for lung cancer and other mediastinal tumors, the radiation dose of 50 Gy and above (2 Gy/time) and 45 Gy (3 Gy/time) were adopted. In group 4, 2% lidocaine was used for local anesthesia in patients undergoing SVC angiography and stent implantation. A 0.035-in. hydrophilic membrane guide wire and a 5-F straight cephalic foramen catheter were introduced through the right femoral vein to enter the SVC under the combination of the two. Contrast agent was injected to develop the SVC. After that, a stent was placed through the inserted 10-F sheath tube to accurately position and release. Balloon dilation was used according to the blockage, and the operation was finished after checking the patency of the vessels and good stent position. There were no accidents during the operation and everything was completed successfully. According to doctors’ experience, warfarin anticoagulants were taken after the operation to prevent thrombosis in the range of 2–3 international standardized ratios. Seven patients received radiotherapy and chemotherapy for anti-tumor therapy after stent implantation. After chemotherapy, one case continued radiotherapy and chemotherapy after stent implantation. The implanted stents included 3 bare stents, 3 covered stents, and 3 wallstent stents, with a diameter of 12–24 mm. Among which, one patient had two covered stents because of the obstruction of SVC and left innominate vein. The total dose of radiotherapy received was above 50 Gy, 2 Gy/time. Chemotherapy drugs included platinums such as pemetrexed, etoposide, gemcitabine, and paclitaxel. Liquid infusion was carried out through lower limb veins, and intensity-modulated radiation therapy was used as radiotherapy. In addition, all patients were treated with diuretics and glucocorticoids to relieve edema.

### Outcome event

The primary outcome event was organ failure and death due to tumor progression, and the secondary outcome event was recurrence of SVCS.

### Follow-up method

The general information, treatment methods, and adverse reactions of patients were obtained from the hospital case database, and the current situation of patients was followed up by telephone. According to the follow-up rate = the actual number of cases followed up during the period (the number of cases to be followed up during the period − the number of people lost to follow up)/the number of cases to be followed up during the period × 100%, the follow-up rate = (48 − 1)/48 × 100% = 97.9%.

### Statistical analysis

Statistical analysis of the collected data was performed using SPSS 25 software. The patients were divided into four groups according to the treatment methods, and the differences in general information and curative effect of the four groups were compared. Multi-group comparison among classifying variables such as gender, smoking history, drinking history, liver function, and occurrence of esophageal mucosal injury was analyzed by chi-square test. One-way analysis of variance was used for the multi-group comparison of measurement data such as age. Kruskal-Wallis rank sum test was used to compare the grade data of myelosuppression, anemia, degree of digestive tract reaction, and treatment effect among multiple groups. If there was statistical significance, post hoc test was used to compare differences between groups. The overall survival rate was estimated using GraphPad Prism, then a survival curve was completed, and the log-rank test was used to analyze differences between groups. Cox proportional hazards model was used to analyze the factors affecting survival time. The results were considered statistically significant when P < 0.05.

## Results

### Comparison of general data

There were no significant differences in gender (P = 0.065), age (P = 0.172), smoking history (P = 0.368), and drinking history (P = 0.138).

### Comparison of therapeutic effect

There were no accidents in the patients receiving the comprehensive treatment during stent implantation. Postoperatively, all patients achieved smooth blood flow of SVC, and the symptom remission rate was 62.5% within 24 h, and the symptom remission rate of SVCS was 100% within 48 h. According to the clinical effective rate = (CR + PR)/(CR + PR + SD + PD), the majority of patients receiving different treatment regiments had good results. The clinical effective rates of group 1, group 2, group 3, and group 4 were 50%, 62.5%, 54.5%, and 50%, respectively (see Table 3). Chemotherapy alone seemed to be able to relieve symptoms more quickly, but there was no statistical significance by post hoc test (H = 0.107, P = 0.991).

**Table 3:** Comparison of therapeutic effect (n, %)

Group	Clinical effective rate (%)	CR	PR	SD	PD
Group 1 (n = 10)	50	0	5	5	0
Group 2 (n = 8)	62.5	0	5	2	1
Group 3 (n = 22)	54.5	1	11	6	4
Group 4 (n = 8)	50	0	4	4	0

Note: Clinical effective rate = (CR + PR)/(CR + PR + SD + PD). *Group 1* radiotherapy group, *Group 2* chemotherapy group, *Group 3* combined radiotherapy and chemotherapy group, *Group 4* comprehensive treatment group, *CR* complete remission, *PR* partial remission, *SD* stable degree, *PD* progression degree

### Comparison of side effects

There were slight differences in the incidence of side effects among different groups, but the overall difference was not statistically significant (all P > 0.05). The effects of radiotherapy alone on the esophageal mucosa of patients were mainly dysphagia and sore throat, which occurred in one case (10%) during the treatment. In group 3, 45.5% of patients had sore throat and dysphagia, which was better than that in group 4 (25%), but not found in group 2. The incidence of myelosuppression (70%) in group 1was slightly lower than that in group 2, group 3, and group 4 (88%, 86.4%, and 88%, respectively). There were 2 cases of grade III or above myelosuppression in group 2, 2 cases in group 3, and 3 cases in group 4. Almost all the patients presented anemia of different degrees, group 1 (8 cases, 80%), group 2 (7 cases, 87.5%), and group 3 (21 cases, 95.4%) were mild and moderate anemia, while 1 in 8 cases (12.5%) of anemia in group 4 was severe. The digestive tract reactions mainly appeared in group 2 (8 cases, 100%), group 3 (17 cases, 77.3%), and group 4 (5 cases, 62.5%). Liver function abnormalities were also found only in group 2, group 3, and group 4, and the probability of liver function abnormality in group 2 (1 case, 12.5%) was significantly lower than that in group 3 (10 cases, 45.5%) and group 4 (2 cases, 25%). In the group 4, 2 cases (25%) who received stent placement in the SVC developed stent thrombosis despite warfarin anticoagulation. Mild anemia occurred in 1 case (50%) of the 2 thymic tumors in group 1. Grade I digestive tract reaction occurred in 1 case (100%) of group 2. One patient (100%) in group 3 developed only grade I digestive tract reaction and mild anemia (see Table 4).

**Table 4:** Comparison of side effects

	Group 1	Group 2	Group 3	Group 4	P
Myelosuppression (%)					
I	5 (50)	4 (50)	6 (27.3)	2 (25)	0.145
II	2 (20)	1 (12.5)	11 (50)	2 (25)	
III	0 (0)	1 (12.5)	1 (4.5)	3 (37.5)	
IV	0 (0)	1 (12.5)	1 (4.5)	0 (0)	
Anemia (%)					
Mild	5 (50)	3 (37.5)	16 (72.7)	5 (62.5)	0.601
Moderate	3 (30)	4 (50)	5 (22.7)	2 (25)	
Severe	0 (0)	0 (0)	0 (0)	1 (12.5)	
Digestive tract reactions (%)					
I	0 (0)	2 (25)	6 (27.3)	3 (37.5)	0.005*
II	0 (0)	6 (75)	11 (50)	2 (25)	
Esophageal mucosal injury (%)	1 (10)	0 (0)	10 (45.5)	2 (25)	0.131
Liver function abnormality	0 (0)	1 (12.5)	10 (45.5)	2 (25)	0.389

*P = 0.005 was because the radiotherapy group was included in the analysis, and pairwise comparison showed that the difference between the other three groups was not statistically significant. *Group 1* radiotherapy group, *Group 2* chemotherapy group, *Group 3* combined radiotherapy and chemotherapy group, *Group 4* comprehensive treatment group

### Comparison of survival rate

**Fig. 1:**
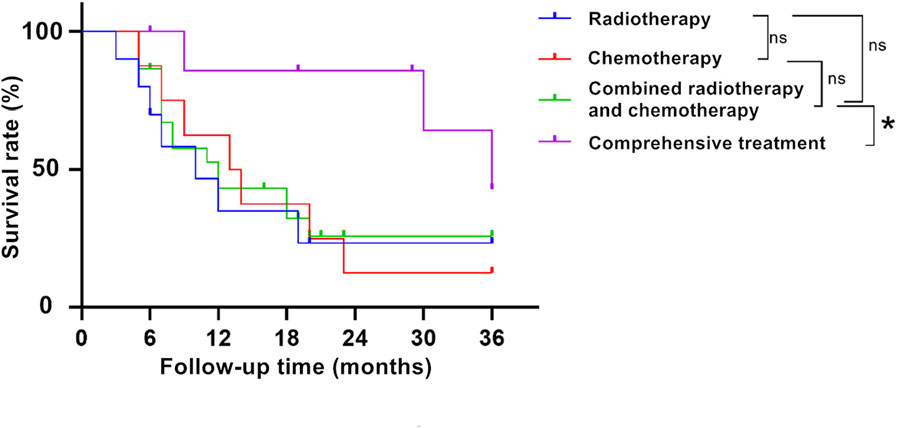
Comparison of survival rate. ns indicated that the difference between the two groups was not statistically significant, while asterisk indicated that the difference between the two groups was statistically significant. The initial event was diagnosis and treatment started, and the endpoint time was death

The median survival times of group 1, group 2, group 3, and group 4 were 10 months, 13.5 months, 12 months, and 36 months, respectively. Among the four survival curves, the survival rate of group 4 was slightly better than that of the other three groups (P = 0.18) (see Fig. 1). By comparing the survival rate, it was found that group 4 was better than group 3 (P = 0.039). According to the analysis, general information such as age and gender had no significant effect on survival time, while tissue type was closely related to survival time (P = 0.005) (see Table 5).

**Table 5:** Relationship between tissue type and survival rate

	Unadjusted (n = 48)	Adjusted*(n = 48)
	HR (95% CI)	HR (95% CI)
Histology types	0.609 (0.434, 0.855)	0.542 (0.355, 0.830)
P	0.004	0.005

*Included age, gender, tissue type, smoking history, and drinking history

## Discussion

Superior vena cava (SVC) is a short and thick vessel about 7 cm long, located in the right front of the upper mediastinum and between the sternum and the spine. It is composed of the left and right brachiocephalic trunk veins and converges behind the junction of the right first costal cartilage and sternum. It is surrounded by thoracic aorta, right main bronchus, trachea, and mediastinal lymph nodes [13, 14]. SVC collects the blood from the head, face, neck, upper limbs, and upper chest and flows down to the lower edge of the third sternal joint and into the right atrium. The wall is thin and the internal pressure is small. Therefore, when lung malignant tumor, mediastinal mass, and lymph node enlargement occur, it will compress and invade the SVC, causing the jugular venous pressure to increase to 20–40 mmHg [15–17]. SVCS due to benign etiology often leads to clinical manifestations such as cough. SVCS caused by malignant etiology can cause edema in the head, face, neck, and upper limbs of patients; dilation of jugular vein and upper chest wall vein; cough; and hoarseness. In severe cases, laryngeal edema may occur, resulting in dyspnea, and even life-threatening while leading to brain edema, seriously affecting the quality of life of patients [18–20].

The comparative analysis showed that the clinical control effect of chemotherapy combined with radiotherapy and chemotherapy alone were slightly better than those of radiotherapy alone and comprehensive treatment (P = 0.991). The incidences of esophageal mucosal injury (P = 0.131), myelosuppression (P = 0.145), and anemia (P = 0.601) caused by radiotherapy were lower than those of the other three groups, while the incidences of digestive tract reaction (P = 0.005, P = 0.005 indicated there was no significant difference in Table 4) and liver function injury (P = 0.389) caused by combined radiotherapy and chemotherapy and comprehensive treatment were higher than those caused by chemotherapy alone (P > 0.05). The survival rates of patients treated with radiotherapy, chemotherapy, and combined radiotherapy and chemotherapy were almost the same. The survival rate of patients treated with comprehensive treatment was significantly better than that of the former three. After comparative analysis, the difference between combined radiotherapy and chemotherapy and comprehensive treatment was statistically significant (P = 0.039). Myelosuppression occurred in nearly 90% of patients receiving radiotherapy and chemotherapy at doses of less than 70 Gy [21]. In the study of radiotherapy and chemotherapy alone, the incidence of mucosal injury was about 26% [22]. In this study, the incidence of myelosuppression in group 3 and group 4 were 86.3% and 87.5%, respectively, and the incidences of mucosal injury were 45.5% and 25%, respectively. In addition, there was no significant difference in the probability of adverse reactions between group 3 and group 4, which ensured the smooth progress of subsequent treatment after stent implantation. In a single-center study of thymic epithelial tumors, for large tumors causing SVCS that could not be completely resected by surgery, whether minimally invasive or open surgery, 40 patients enrolled had a 30% incidence of postoperative complications of Clavien-Dindo grade III or higher, and 1 patient died after surgery. Severe complications increased the risk of death by 4.904 times [23]. The 4 thymic tumor patients enrolled in this study only showed grade I digestive tract reaction and mild anemia. Therefore, for thymus tumors complicated with SVCS, it is a good choice to choose radiotherapy, chemotherapy, or chemoradiotherapy for tumor reduction.

In the present study, all patients who received stent implantation to relieve SVCS achieved smooth flow of SVC, with a 100% symptom remission rate within 48 h. Compared with percutaneous transluminal angioplasty (PTA), in a study that enrolled patients (14.3% had malignant etiology causing SVCS), 7% of the patients received PTA treatment, 57% of the patients received PTA and stent implantation, 21% received PTA and thrombolysis, and 7% received thrombolysis, reaching 86% of the symptom remission rate and 90% of the initial patency rate [24]. In addition, the persistence of SVC with only PTA to open the occlusion is poor. For malignant SVCS, PTA combined with stent implantation may achieve more effective and lasting clinical effects than PTA alone. The use of autologous pericardial tissue instead of SVC is also used to alleviate malignant SVCS, and its advantages of high safety and less postoperative complications make this method have great development potential [25, 26]. Although limited by the size of the pericardial tissue, the use of autologous tissue does not cause immune rejection, and the probability of thrombosis is greatly reduced.

In addition, surgery can be used as a treatment for SVCS, as well as to remove the lesions that cause SVCS. In a study of SVCS caused by nodular lymphadenopathy, patients were treated with vascular reconstruction surgery with satisfactory results [27]. However, for patients with malignant SVCS, the basic condition is poor, so endovascular therapy is more acceptable to patients, and endovascular therapy is combined with anti-tumor therapy to control the disease progression [28].

According to statistical analysis, the median survival time of each group in this study was 10 months, 13.5 months, 12 months, and 36 months, respectively. Pairwise comparison showed that the long-term survival rate of comprehensive treatment was more objective than that of combined radiotherapy and chemotherapy. This may be due to the different types of tissues included in the two groups. The ratio of lung cancer to other tumors was 17:5 in group 3 and 1:1 in group 4. The median survival time of patients with lung cancer after stent implantation in the SVC after chemotherapy and radiotherapy can reach 7 months (1–18 months) [29]. In addition, in the multi-tissue-type study of stent implantation and chemotherapy, the median survival time for SCLC and NSCLC was 95 days and 121 days, and the median survival time for other cancer patients after stent implantation was 143 days. The prognosis of lung cancer was worse than that of other tissue-type tumors [30]. After stent implantation, a series of symptoms caused by patients’ discomfort disappear rapidly, and patients will have a high acceptance of the follow-up anti-tumor treatment [31].

However, there are some limitations in this study. Firstly, due to the small sample size and short observation time, a large-sample, multi-center, and long-term observation study is needed; secondly, the selected cases were from the same hospital and the number was too small to make a firm judgment on each treatment, and a further study is necessary. However, the study confirmed that stent implantation does not affect the patient’s tolerance and long-term survival for subsequent radiotherapy and chemotherapy. Radiotherapy and chemotherapy, whether used alone or in combination, had acceptable side effects and achieved satisfactory efficacy and survival time.

## Conclusions

In conclusion, for patients with severe symptoms or long predicted survival of SVCS, stent implantation was feasible for rapid relief of symptoms and provided conditions for subsequent treatment; if mild symptoms were tolerable, patients could choose radiotherapy and chemotherapy or combined radiotherapy and chemotherapy to treat the primary tumor while alleviating symptoms, so as to reduce the cost of treatment.

## Data Availability

All data generated or analyzed during this study are included in this published article.

## Abbreviations

SVCS: Superior vena cava syndrome
NSCLC: Non-small cell lung cancer
SCLC: Small cell lung cancer
NHL: Non-Hodgkin’s lymphoma
SVC: Superior vena cava
CR: Complete remission
PR: Partial remission
SD: Stable degree
PD: Progression degree
PTA: Percutaneous transluminal angioplasty
